# Modified dipeptide based nanospheres as a potent adjuvating delivery system for recombinant vaccines

**DOI:** 10.3389/fddev.2023.1135209

**Published:** 2023-04-26

**Authors:** Saikat Biswas, Nitin Yadav, Anjali Somanathan, Paushali Mukherjee, Virander Singh Chauhan

**Affiliations:** ^1^ Molecular Medicine Group, Molecular Medicines International Centre for Genetic Engineering and Biotechnology, New Delhi, India; ^2^ School of Biotechnology, Jawaharlal Nehru University, New Delhi, India; ^3^ Multi Vaccines Development Program (MVDP), New Delhi, India

**Keywords:** biomaterial, dipeptide, nano delivery, adjuvant, malaria, recombinant vaccine, humoral and cellular response

## Abstract

Recombinant protein vaccines offer an advantage without a safety risk in eliciting desired humoral and cell-mediated immune responses against infectious diseases. But one of their disadvantages is their low immunogenicity, thus requiring adjuvants that augment their immunogenicity. It is necessary to explore new technology that could provide a non-toxic, biodegradable, and biocompatible delivery system with adjuvant characteristics and nanotechnology provides an excellent platform for nanomaterial-based vaccine adjuvants. Here, we have synthesized a modified dipeptide, Arg-α, *β*-dehydrophenyalanine (RΔF) containing ΔF at its C-terminal, and characterized it using reversed-phase high-performance liquid chromatography (RP-HPLC) and mass spectrometry techniques. RΔF upon its self-assembly to spherical nanoparticles (NPs) efficiently condensed a recombinant *Plasmodium falciparum* surface protein, histidine-tagged MSPFu24 (Fu24H). The morphological characteristics of the nanoparticle formulation was characterized using TEM. RΔF NPs and RΔF-Fu24H complex showed excellent *in vitro* biocompatibility toward two mammalian cell lines and human red blood cells (RBCs). Furthermore, mice treated with R∆F NPs showed histological and haematological properties similar to the untreated control group which indicated their very high *in vivo* biocompatibility. Mice treated with RΔF-Fu24H nanoformulation induced a high titers of anti-Fu24H specific antibodies and showed a mixed Th1 and Th2 profile, comparable to the FDA-approved adjuvant Alhydrogel^®^. The sera from immunized mice inhibited the erythrocyte invasion activity of *P. falciparum’s* laboratory line 3D7 *in vitro* which was comparable to that of Alhydrogel^®^. The present study suggests that the highly biocompatible dipeptide-based nanoparticle formulation can further be developed and used in clinic as a promising antigen delivery platform to elicit immune responses.

## 1 Introduction

Recombinant protein vaccines, expressing one or multiple defined antigens to induce immunity against the pathogen, are safer, tolerable, and less reactogenic than many attenuated and inactivated vaccines in clinical practice. Despite being safer, recombinant proteins are poor immunogens and require adjuvants added to the vaccine formulation to enhance the immune responses and provide greater defense against the target pathogen (Miyaji et al., 2011; Reed et al., 2013). Adjuvants act by enhancing the strength, breadth, and longevity of the innate immune response and thereby triggering a specific-and long-lasting humoral and cellular immune responses (Irvine et al., 2013; Welch et al., 2018; Arunachalam et al., 2021; Reyes and Patarroyo, 2023). Use of appropriate adjuvants to enhance immunogenicity can decrease the amount of antigen required for each vaccine dosage (antigen sparing) and also reduce the number of vaccine doses required to achieve sufficient protection (dose sparing) (Coffman et al., 2010; Reed et al., 2013; Magiri et al., 2018). Adjuvants can be grouped according to their mechanism of action, dividing them into two main categories: delivery systems (antigen carriers) and immune potentiators, although many adjuvants function as both. Due to safety and tolerability issues, only a few adjuvants are available for clinical use but many of these stimulate a suboptimal level of the adaptive immune response (Eisenbarth et al., 2008; Marrack et al., 2009). The adjuvants in licensed human vaccines include aluminum salts (alum), mineral oil-in-water emulsions such as MF59 and AS03, Toll-like receptor (TLR) agonists (CpG or monophosphoryl lipid A adsorbed on aluminum salts as in AS04 or combination of immunopotentiators (QS-21 and MPL) in AS01, Matrix-M adjuvant and Advax adjuvant (Facciolà et al., 2022; Reyes and Patarroyo, 2023).

Nanoparticles (NPs) are a diverse group of nanosized materials with novel physicochemical characteristics that have immunostimulatory effects. Recently, the use of NPs as potential delivery vehicles for vaccine antigens which can both stabilize vaccine antigens and act as adjuvants (also referred to as nano-adjuvants) has attracted attention from nanobiotechnology, pharmacy, and immunology. As carriers and/or adjuvants, nanoparticles (NPs) have a number of significant advantages over conventional adjuvants, including a reduction in the rate of antigenic degradation, an increase in antigen stability, an increase in the therapeutic efficacy and immunogenicity of vaccines, a facilitation of phagocytosis and quick processing by antigen-presenting cells (APCs), and an improvement in cellular membrane penetrability (Bezbaruah et al., 2022). Many different types of nanoparticles, including inorganic and polymeric nanoparticles (poly (lactic-co-glycolic) acid (PLGA), polylactic acid (PLA), polyethylene glycol (PEG), virus-like particles (VLPs), liposomes. Other nanoparticle systems include self-assembled peptides, protein and inorganic NPs like albumin, gold, carbon, chitosan and mesoporous silica have been investigated as potential antigen carriers for vaccines. These NPs offer several advantages including biocompatibility, easy preparation, controllable sizes (He et al., 2010; Morachis et al., 2012; Liu et al., 2013; Niikura et al., 2013; Shang and Nienhaus, 2017; Roma-Rodrigues et al., 2019).

Peptide-based nanomaterials have recently gained attention as delivery systems for biomolecules such as drugs, siRNAs, subunit vaccines, etc. (Rudra et al., 2010; Panda and Chauhan, 2014; Friedrich et al., 2016; Habibi et al., 2016; Song et al., 2019). They are easy to synthesize and can self-assemble into a variety of nanostructures, including nanofibers, nanovesicles, nanotubes, nanomicelles, and hydrogels. NPs have good biocompatibility and biodegradability, less systemic and local toxicity, improved antigen-presenting cell uptake, and improved biological activity. In a more recent technique, immunogenic structures made of self-assembling peptides were built using oligomerization motifs (Lupas and Gruber, 2005; Eskandari et al., 2017; Negahdaripour et al., 2017). However, short half-life due to enzymatic degradation is one drawback of peptide-based drugs and drug delivery vehicles under *in vivo* conditions (Bruno et al., 2013). We previously reported dipeptides containing an unnatural amino acid, α, *β*-dehydrophenylalanine (∆F) residue self-assemble into nanoparticles with characteristic shapes and sizes, and successfully utilized for the delivery of drugs, plasmid DNA and oligonucleotides both *in vitro* and *in vivo* (Mishra et al., 2008; Alam et al., 2012; Panda et al., 2013a; Thota et al., 2016; Yadav et al., 2020; Yadav et al., 2022). The presence of ∆F induces conformational constraint in the peptide backbone and, at the same time, confers resistance to enzymatic action (Panda et al., 2011; Panda et al., 2013b; Varshney et al., 2018). Out of a panel of modified dipeptides, a positively charged dipeptide, RΔF forms highly stable nanospheres, can condense plasmid DNA, protect it from enzymatic degradation, and deliver it efficiently (Panda et al., 2013b; Varshney et al., 2018; Yadav et al., 2022).

In this study, we wanted to take advantage of the delivery potential of RΔF NPs to effectively deliver recombinant malaria vaccine, histidine-tagged PfMSPFu24 (Fu24H), and compare the humoral and cellular responses in comparison to the FDA-approved adjuvant Alhydrogel^®^. We have already reported the development of histidine-tagged PfMSPFu24 recombinant fusion chimaera (Mazumdar et al., 2010; Gupta et al., 2014). The recombinant protein comprises conserved regions of *Plasmodium falciparum* Merozoite Surface Protein 1 (PfMSP-1) and Merozoite Surface Protein 3 (MSP-3). The 19 kDa region of PfMSP-1 (PfMSP-1_19_) was fused to the 11 kDa region of PfMSP-3 (PfMSP-3_11_), which contains both T-helper (Th) epitopes and B cell epitopes that are targets of antibody-dependent cellular inhibition (ADCI) (Mazumdar et al., 2010; Gupta et al., 2014).

The results showed that RΔF NPs may be suitable for the delivery of subunit-based immunogens and induced a significant humoral and cellular response comparable to that of the traditionally used Alhydrogel^®^ adjuvant. The RΔF NPs may be further investigated as a novel vaccine delivery platform for successful delivery and improving the recombinant vaccine’s immunogenicity.

## 2 Materials

Boc-Arg (pbf)-OH was purchased from Iris Biotech GmbH, Germany. Tetrahydrofuran (THF), N-methyl morpholine (NMM), isobutyl chloroformate (IBCF), DL-3-phenylephrine hydrate, sodium hydroxide (NaOH), sodium chloride (NaCl), sodium bicarbonate, trifluoroacetic acid (TFA), 4-(2-hydroxyethyl)-1-piperazineethanesulfonic acid (HEPES), Triisopropylsilane, anhydrous sodium acetate and dimethyl sulphoxide (DMSO), Secondary antibody (A4416) were obtained from Sigma-Aldrich, St. Louis, Missouri, United States. Ethyl acetate and dimethylformamide (DMF) were obtained from Spectrochem, Mumbai, India. Acetic anhydride was purchased from SD fine Chem Limited, Mumbai, India. Anhydrous sodium sulfate, methanol, HPLC grade acetonitrile, 1-propanol, and anhydrous diethyl ether, DAPI were purchased from Merck, Darmstadt, Germany. ELISA kits were purchased from Invitrogen, Waltham, Massachusetts, United States. Dulbecco’s modified eagle’s medium (DMEM) was provided by Gibco, Waltham, Massachusetts, United States. Citric acid, Fetal bovine serum (FBS), 3-(4, 5-dimethylthiazol-2-yl)-2,5-diphenyltetrazolium bromide (MTT reagent), endotoxin-free water, trypsin EDTA, phosphate buffer saline (PBS), gentamycin and trypan blue were obtained from Himedia, Mumbai, India. Confocal dishes were purchased from Gräfelfing, Bayern. IFN γ, IL-2 ELISA kits were purchased from Invitrogen, Waltham, Massachusetts, the United States, and TNF-α, IL-12, IL-6, IL-10, and IL-4 ELISA kits were purchased from R&D Systems, Minneapolis, United States.

## 3 Methods

### 3.1 Synthesis and characterization of R∆F

Dipeptide, R∆F was synthesized using the solution-phase peptide synthesis method (Panda et al., 2013a; Varshney et al., 2018). The detailed procedure is provided in Supplementary Text S1. The synthesized R∆F was characterized using RP-HPLC (Shimadzu) on a C-18 column (Phenomenex) using a 5%–95% gradient (1 mL/min for 45 min) of acetonitrile-water containing 0.1% TFA (degassed and filtered) and electron spray ionization mass spectrometry (Applied Biosystems QStar (Q-TOF)).

### 3.2 Self-assembly of R∆F into nanoparticles

The self-assembly of R∆F was studied at a concentration of 1–10 mg/mL. 1 mg R∆F was dissolved in 30–50 μL of hexafluoroisopropanol (HFIP) and the sample was sonicated for 15 min in a bath sonicator. To the above solution, 970–950 µL of Milli Q water (filtered, sterile) was added to make 1 mg/mL NPs solution and followed by incubation at room temperature for 60 min.

### 3.3 Characterization of R∆F nanoparticles using dynamic light scattering (DLS)

Hydrodynamic size measurements for R∆F nanoparticles were carried out using a zeta sizer (Malvern Zetasizer Nano Series Nano ZS90) at 37°C with laser (10 mW HeNe laser, 633 nm) and the experimental procedures reported earlier (Panda et al., 2013b; Thota et al., 2016; Varshney et al., 2018; Yadav et al., 2022).

### 3.4 Expression and characterization of Fu24H

The expression and purification of Fu24H have been described earlier (Mazumdar et al., 2010; Gupta et al., 2014). Briefly, Fu24H was cloned and expressed in an *Escherichia coli* system. The fusion protein was purified using Ni^2+^ affinity chromatography followed by anion-exchange chromatography. Protein purity was analyzed by RP-HPLC on a C-18 column (Phenomenex) using a 5%–95% gradient (1 mL/min) of acetonitrile-water containing 0.1% TFA and 15% SDS-PAGE electrophoresis, followed by a western blot. For the western blot analysis, Fu24H was separated on 15% SDS-PAGE and transferred to a nitrocellulose membrane. After blocking with 5% skimmed milk in PBS at 37°C for 2 h, the blot was first incubated with a PfMSPFu24-specific antibody and then with an HRP-conjugated anti-mouse IgG secondary antibody (Sigma, St. Louis, MO, United States) for 1 h. After washing, the bound protein in the immunoblot was detected with Pierce^™^ ECL Western Blotting Substrate (Thermo Scientific, Rockford, IL, United States) and 1 μL/mL H_2_O_2_ in PBS.

### 3.5 Entrapment of Fu24H into R∆F NPs

The Fu24H was mixed with preformed R∆F NPs at a ratio of 1:10; 1:20; 1:30; 1:40; 1:50 (R∆F: Fu24H, w/w), followed by overnight incubation at 25°C with gentle mixing. The reaction mixture was ultracentrifuge at 1.2 lakh rpm for 3 h at 25°C. To measure entrapment efficiency, an aliquot of 25 μL supernatant was transferred to a 96-well plate having BCA reagent (Pierce^™^ BCA Protein Assay Kit, Thermo Scientific, United States) and the reaction mixture was mixed well and incubated for 30 min at room temperature. Absorbance was measured at 562 nm using UV-visible spectroscopy (VersaMax, Molecular Devices, Sunnyvale, California, United States), and entrapment efficiency was calculated as follows: % Entrapment efficiency = Amount of Fu24H in R∆F NPs/Total amount of Fu24H added x 100.

### 3.6 Characterization of R∆F NPs and R∆F-Fu24H complex using transmission electron microscopy (TEM)

The size and morphology of the R∆F NPs and R∆F-Fu24H complex were studied using TEM. An aliquot of 20 µL of R∆F NPs and R∆F-Fu24H complex was loaded onto carbon-coated copper grids, followed by incubation for 45 min at room temperature. The excess sample from the grids was removed using a Whatman filter paper, and the samples were negatively stained with 1% uranyl acetate (filtered with a 0.22 µm filter). The grids were visualized using the 120 kV mode of TEM (Tecnai 12 BioTWIN, FEI Netherlands) and micrographs were analyzed using Analysis II (Megaview, SIS, Germany) software.

### 3.7 Cell lines culture

HEK 293T and WRL 68 cell lines were maintained in DMEM with 3.7 g sodium bicarbonate, 3.7 g HEPES, 10% FBS, and 0.1% penicillin-streptomycin. All cell lines were maintained in a humidified incubator at 37°C with 5% CO_2_ and passaged at a confluency of 70%–80%.

### 3.8 Cytotoxicity of R∆F NPs and R∆F-Fu24H complex in different cell lines

For toxicity studies, 5 × 10^3^ cells/well (HEK 293T and WRL 68 cell lines) were seeded in a flat-bottomed 96-well plate and incubated at 37°C with 5% CO_2_. After 12 h, cells were treated with RΔF NPs, R∆F-Fu24H complex at different concentrations (10 μg/mL to 50 μg/mL) for 24 h, followed by the addition of 20 μL of MTT reagent (5 mg/mL in PBS). After 3 h of incubation, media was removed and formazan crystals were dissolved in 100 µL of dimethyl sulfoxide (DMSO). The cell viability was measured on a microplate reader VersaMax ELISA reader (Molecular Devices, Sunnyvale, California, United States) at 570 nm and cell viability (%) was calculated using the equation: (Absorbance of treated cells/Absorbance of control) x100.

### 3.9 *In vitro* hemolysis of R∆F NPs and R∆F-Fu24H complex

Human O+ Red blood cells (RBC) stored in 10% citrate-phosphate-dextrose were obtained from the Rotary Blood Bank (New Delhi, India). RBCs were washed three to five times using phosphate-buffered saline (PBS) and centrifuged at room temperature at 1,500 g for 10 min. The packed cell volume obtained was used to make a 10% (v/v) suspension in PBS. Aliquots of 100 μL of the packed RBC suspension were transferred to a 96-well microtiter plate (Corning, New York, United States) and 100 μL of different concentrations of R∆F NPs alone (10–50 μg/mL) and R∆F-Fu24H complex (25–50 μg/mL) were added to the respective wells. The plate was incubated at 37°C for 3 h and then centrifuged at room temperature at 1,500 g for 10 min. An aliquot of 100 μL supernatant was transferred to a new microtiter plate and absorbance was measured at 540 nm using a microplate reader VersaMax ELISA reader (Molecular Devices, Sunnyvale, California, United States) to measure RBC lysis. Cells incubated with PBS alone acted as the negative control, and RBCs treated with 0.2% Triton X-100 were used as a positive control.

### 3.10 *In vivo* acute toxicity of R∆F NPs

Mice were intravenously administered with R∆F NPs at 60 mg/kg concentration and continuously monitored for any behavioural and body weight changes. For the *in vivo* toxicity study, a dose of 60 mg/kg for R∆F NPs was used which was 1.2 times excess to the dose utilised for *in vivo* immunisation studies (50 mg/kg). Post NPs administration, animals were kept under observation for 30 min. After 6 h of dosing, mice were euthanized using CO_2_ inhalation and blood with other major organs including liver, kidney, spleen, and heart were collected for further analysis by complete blood count (CBC) and histopathology studies. All observations were systematically recorded and animals with any moribund condition or severe pain or enduring signs of severe distress were humanely killed without delay.

### 3.11 *In vitro* immunogenicity of R∆F NPs

DCs and T cells (>90% pure) were purified from splenocytes of BALB/c mice (6–8 weeks) using 30% BSA in PBS, anti-CD11c-biotin and anti-CD3-biotin (from Biolegend) antibodies, and Streptavidin MicroBeads as described earlier as per the manufacturer’s instructions (Miltenyi Biotec) (Tamura et al., 2005; Verma et al., 2021). DCs and T cells (>90% pure) fractions purified from separate spleens were incubated in the ratio of 1:5 (1 × 10^4^ DCs and 5 × 10^4^ T cells) in the U bottom 96 well plate. After 12 h of incubation, the cells were stimulated with a serial dilution of R∆F NPs from 5 μg/mL to 20 μg/mL and MAGE 3 at 10 μg/mL, which was used as a positive control. After 72 h, the cells were centrifuged at 2000 rpm for 10 min at 4°C. Supernatants were collected, and T cells responses were measured by quantifying IFN-γ using an enzyme-linked immunosorbent assay (ELISA) according to the manufacturer’s instructions (R&D Systems, United States).

### 3.12 Immunization of mice with R∆F-Fu24H complex

BALB/c mice were obtained from the Jackson Laboratory and housed in the animal house facility of the International Centre for Genetic Engineering and Biotechnology (ICGEB) under pathogen-free conditions. 6–8 weeks old female mice, were randomly allocated into two groups with 6 mice/group. Mice were immunized with an aliquot of 25 µg of Fu24H formulated with Alhydrogel^®^ and R∆F NPs. As reported in our earlier published work, the dose of 25 µg of the protein antigen in mice has been optimised and has been used in different formulations, including Alhydrogel and nanostructures like FΔF and LΔF hydrogels (Mazumdar et al., 2010; Gupta et al., 2014; Anand et al., 2021). Animals were immunized intramuscularly at 0 days, 28 days, and 56 days. Sera were collected before immunization at day −2 and at day 70 after the last immunization.

### 3.13 Enzyme-linked immunosorbent assay (ELISA) of sera samples

Sera collected from individual immunized mice (n = 6/group) were measured for anti-Fu24H antibody response by ELISA. Briefly, flat-bottom 96 well, microtiter ELISA plates (MaxiSorp, Nunc) were coated with Fu24H (2 μg/mL) in a carbonate-bicarbonate buffer and incubated overnight at 4°C. Plates were washed three times with phosphate-buffered saline (PBS) containing 0.05% Tween 20 (PBST) and blocked with 2% skimmed milk powder in PBS (pH 7.2) for 2 h at 37°C. The immune sera were three-fold serially diluted in PBST containing 0.25% skimmed milk powder and added to antigen-coated plates and incubated for 1 h at 37°C. Following three washes with PBST, goat anti-mouse IgG conjugated with horseradish peroxidase (Sigma) was added to the wells and incubated for 1 h at 37°C. After final washings with PBST followed by PBS, 1 mg/mL o-phenylene diamine dihydrochloride (Sigma) as chromogen and hydrogen peroxide as substrate were added to the plate. The color was allowed to develop for 20 min in dark and the reaction was stopped with 2.0 N sulfuric acid. The absorbance was measured at 490 nm using a VersaMax ELISA reader (Molecular Devices, Sunnyvale, California, United States). End-point IgG titers were established as the mean plus three standard deviations of absorbance of pre-immune sera. The end-point titer was calculated using a 4-parameter curve fitting model (GraphPad Prism Software, version 6.01, San Diego, CA, United States). The endpoint titer is defined as the reciprocal of the highest dilution of a serum that gives an absorbance above the cut-off OD value. And the cut-off value is defined as the mean plus three standard deviations of absorbance of pre-immune sera.

### 3.14 Cytokine analysis of sera samples

Cytokines were quantified in sera collected from individual mice (n = 6/group) before immunization on day −2 and 2 weeks after the last immunization at 70 days. The levels of pro-inflammatory cytokines: IFN-γ, TNF-α, IL-12, IL-6; and anti-inflammatory cytokines: IL-10 and IL-4 in the serum were measured using a capture ELISA according to the manufacturer’s protocol (R&D System, United States). Before the assay, sera samples were diluted 1:4 in sample diluent. Concentrations were determined by generating standard curves for each cytokine using the cytokine standard provided with the kit.

### 3.15 Growth inhibition assay (GIA) *Plasmodium falciparum*

Sera collected from immunized mice (n = 6/group) were tested for their ability to inhibit the *P. falciparum* parasite (3D7 lab strain) replication by an *in vitro* growth inhibition assay (GIA), as described previously (Mazumdar et al., 2010; Gupta et al., 2014; Anand et al., 2021). For the parasite culture, human O+ RBCs were procured from Rotary blood bank, New Delhi, India. *P. falciparum* parasites were cultured in human O+ RBCs and synchronized through treatments with Percoll and sorbitol. For the invasion inhibition assay, synchronized parasites were adjusted to 2% hematocrit and 0.3% parasitemia in a late-trophozoites/early-schizonts stage and incubated in the presence or absence of sera. The sera were added to the parasite culture at a final dilution of 1:10 and incubated for one cycle (44 h post-invasion) under a mixed gas environment (90% N_2,_ 5% CO_2_, 5% O_2_). Parasite growth was assessed by staining parasite-infected RBCs with ethidium bromide (EtBr) at a concentration of 10 μg/mL in PBS and measured by flow cytometry. Invasion inhibition was calculated with respect to pre-immune sera as % inhibition = 1—[(percent invasion from immune sera)/(percent invasion from preimmune sera)] × 100.

### 3.16 Statistical analysis

Data were analysed using GraphPad Prism Software, version 6.01(San Diego, CA, United States). Data are shown as mean ± standard deviation (SD). For antibody titers, the log10-transformation of the titers were statistically compared. Differences between the groups were compared using unpaired Student’s t-test after confirming the assumptions of normality. Differences were considered significant at a *p*-value < 0.05 and n = sample size (biological repeat).

## 4 Results and discussion

### 4.1 Synthesis, characterization, and formation of R∆F NPs

**FIGURE 1:**
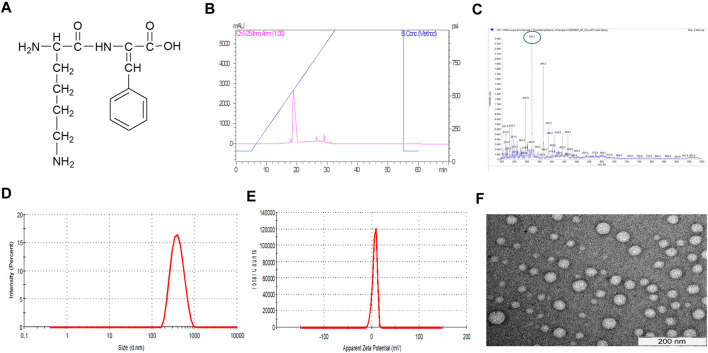
**(A)** Chemical structure of R∆F. **(B)** RP-HPLC profile of R∆F. **(C)** Mass spectrometry (electron spray ionization) profile of R∆F. **(D)** Hydrodynamic diameter of self-assembled R∆F at 1 mg/mL concentration shows the formation of homogeneous NPs (*n* = 5). **(E)** Zeta potential of R∆F NPs using DLS. **(F)** Transmission electron microscopy image of R∆F NPs.

R∆F (Figure 1A) was synthesized using the standard solution-phase synthesis procedure (Supplementary Text S1). The dipeptide was characterized by RP-HPLC and mass spectrometry. RP-HPLC profile of R∆F showed a single peak at 19 min and a purity of more than 95% (Figure 1B). Mass spectrometry analysis indicated that the observed molecular mass of R∆F was 320.2 Da (319.3 Da + 1H^+^) (Figure 1C). Self-assembly of the dipeptide was initiated by solubilising R∆F in HFIP and then adding water to the mixture. R∆F at 1 mg/mL self-assembled to form homogeneous nanospheres having a hydrodynamic diameter of 368.62 ± 108.4 nm with a PDI of 0.22 ± 0.06 as assessed by DLS (Figure 1D). Zeta sizer analysis showed that R∆F NPs acquired a surface charge of ∼5.16 ± 1.01 mV (Figure 1E). TEM images revealed that R∆F NPs formed well-defined spherical structures with a diameter in the range of ∼50–100 nm (Figure 1F).

### 4.2 Characterization of Fu24H

**FIGURE 2:**
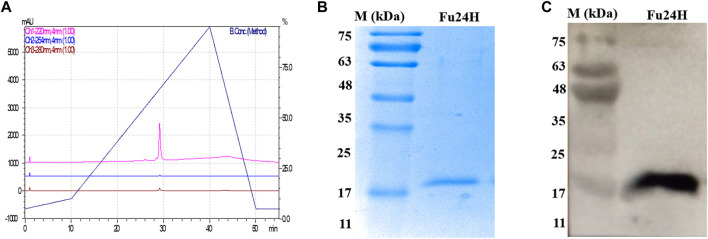
**(A)** RP-HPLC profile of Fu24H. **(B)** 15% SDS-PAGE of Fu24H (M., molecular marker). **(C)** Western blot of Fu24H (M., molecular marker).

The purity of Fu24H was analyzed by RP-HPLC. Fu24H eluted as a single peak at a retention time (RT) of 29 min with more than 98% purity (Figure 2A). Purified Fu24H was observed as a single band on 15% SDS-PAGE and by western blot (Figures 2B, C and Supplementary Figure S1).

### 4.3 Formation and characterization of R∆F-Fu24H complex

**FIGURE 3:**
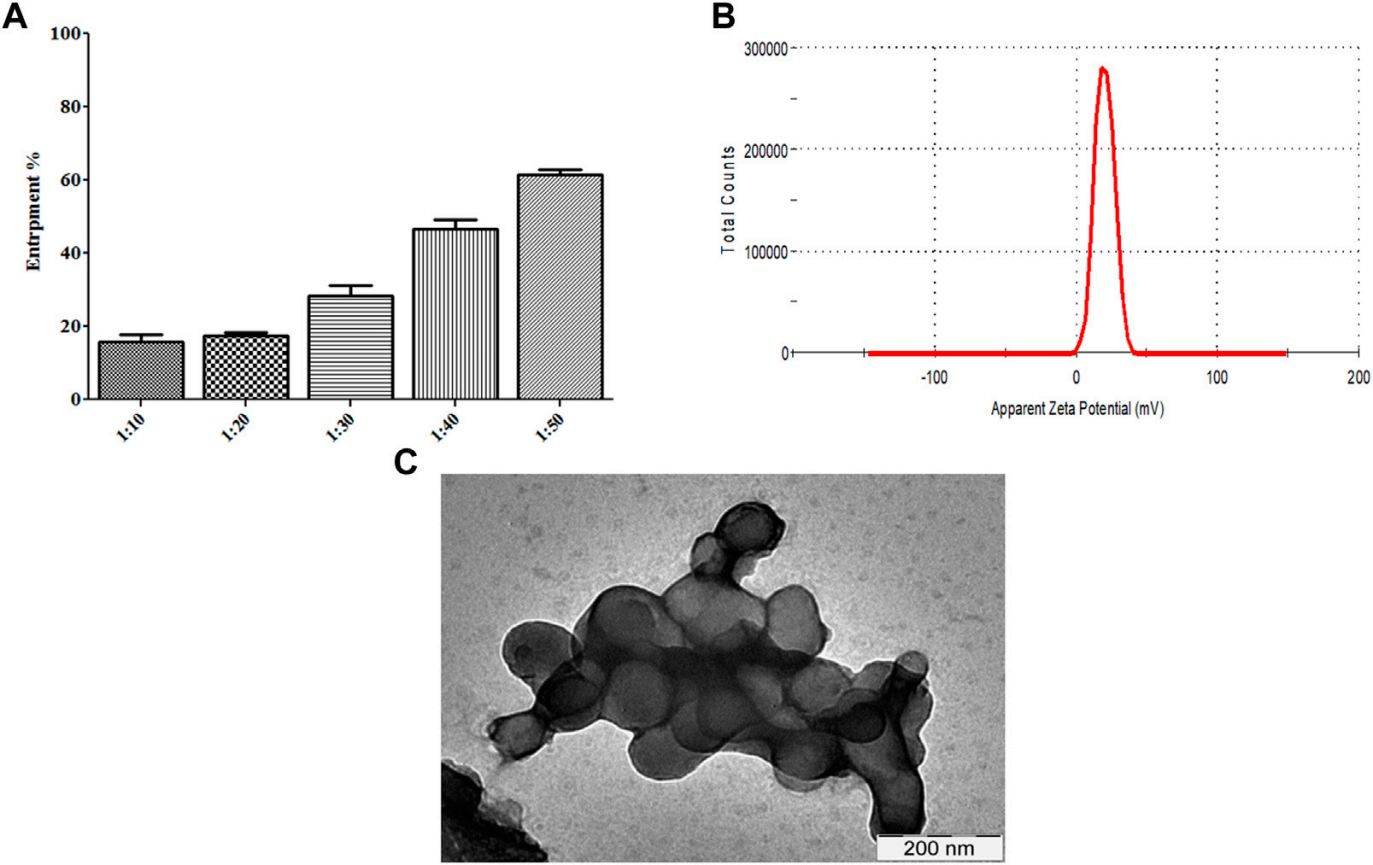
**(A)** Entrapment of Fu24H into R∆F NPs. **(B)** Zeta potential of R∆F-Fu24H complex studied using DLS. **(C)** Transmission electron microscopy image of R∆F-Fu24H complex.

We determined whether R∆F NPs form a stable complex with Fu24H NPs. Fu24H (25 µg) were mixed with different ratios of R∆F NPs (Fu24H: R∆F NPs; 1:10, 1:20, 1:30, 1:40, 1:50; w/w) and incubated overnight at 25°C with gentle mixing. At a ratio of 1:50, (Fu24H: R∆F NPs) optimum entrapment (61.44% ± 1.40%) was observed and this ratio was used in all further experiments (Figure 3A). R∆F-Fu24H complex showed a surface charge of ∼19.87 ± 1.11 mV in the zeta sizer (Figure 3B). Whether the morphology of RΔF nanoparticles changed upon complexation with Fu24H was investigated by TEM. TEM images of RΔF showed no significant morphological changes in the shape after loading with Fu24H antigen (size < 150 nm), but a nanoparticle cluster formation was observed (Figure 3C).

### 4.4 Biocompatibility of R∆F and R∆F-Fu24H complex

**FIGURE 4:**
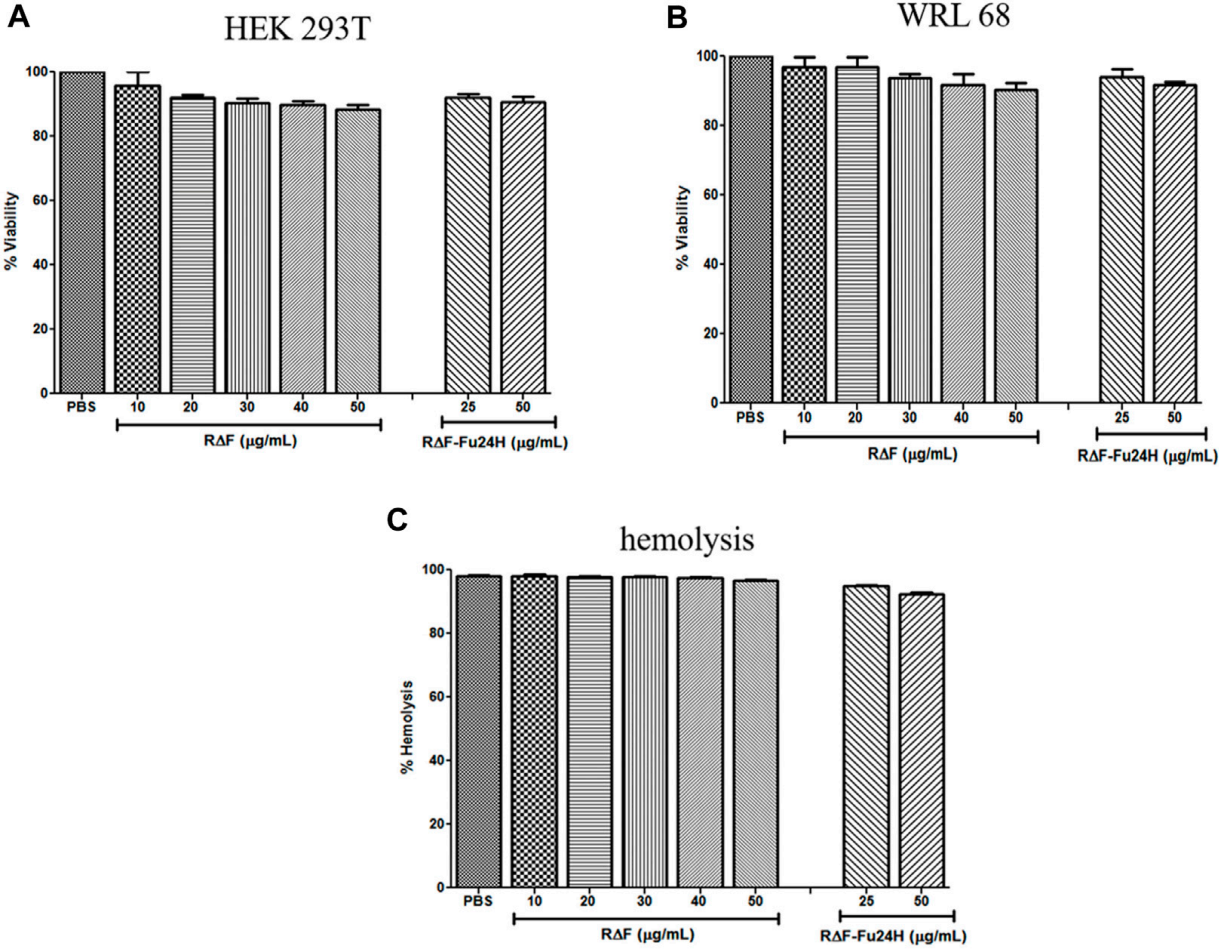
Cytotoxicity of different cell lines after treatment with R∆F NPs and R∆F-Fu24H complex using MTT assay. Percentage viability for cells treated with R∆F NPs and R∆F-Fu24H complex at different concentrations **(A)** HEK 293T and **(B)** WRL 68, and **(C)** Percentage hemolysis caused by R∆F NPs and R∆F-Fu24H complex. Data has been reported as mean ± standard deviation (SD) from three separate experiments (*n* = 3).

After confirming the physicochemical characteristics of R∆F NPs and R∆F-Fu24H complex, we next investigated their potential biological properties, including cellular toxicity, hemocompatibility, and anti-inflammatory effects. The cellular toxicity of R∆F NPs and R∆F-Fu24H complex were tested on different cell lines, HEK 293T, and WRL 68 cells, using the standard MTT assay. No cytotoxicity was observed in any of the cell lines when treated with R∆F NPs alone or with R∆F-Fu24H complex (Figures 4A–C). Since injected nanoparticles would inevitably come into contact with blood, it is critical to assess the potential toxicity of R∆F NPs and R∆F-Fu24H complex on RBCs (Chen et al., 2015). In this context, we investigated whether R∆F NPs and R∆F-Fu24H complex caused hemolysis in RBCs. When used at the highest concentration of 50 μg/mL, both R∆F NPs and R∆F-Fu24H complex resulted in less than 5% hemolysis and caused no damage to RBCs (Figure 4D). This result confirms the hemocompatibility of the nanoparticles. Nanoparticles have been shown to influence inflammatory cell responses. We, therefore, investigated whether R∆F NPs activate an inflammatory response when murine dendritic cells and T cells (DC-T) were cocultured in the presence of RΔF NPs. Our result showed that RΔF NPs did not stimulate production of the inflammatory cytokine IFN-γ in the DC-T coculture (Supplementary Figure S1). These findings indicated the high biocompatibility of R∆F NPs *in vitro*.

### 4.6 *In vivo* toxicity of R∆F NPs

To determine the toxicity of R∆F NPs *in vivo*, mice were injected intravenously with the NPs, and the toxicity was evaluated by analyzing histological and haematological data, as well as animal behavioural patterns. Compared to the PBS-treated control group, the mice treated with R∆F NPs showed no abnormal behaviour. At the end of the experiment, major organs (heart, liver, kidney, and spleen) were harvested, and a microscopic examination of the tissue was performed after staining with hematoxylin and eosin (H&E). Histopathological images revealed there was no tissue damage or abnormalities in mice treated with the NPs compared to control group (Supplementary Figure S2). We further evaluated potential hematotoxicity of the nanoparticles in mice and blood was collected after euthanasia for complete blood count (CBC). CBC is a panel of blood tests used to know important details about the blood’s cellular composition. CBC parameters, such as White blood cells, Red blood cells, Haemoglobin, Haematocrit, Total Leucocyte Count, Platelet Count, Neutrophils, and Lymphocytes, were all within the normal range for NPs and PBS groups (Table 1). Results of *in vivo* toxicity study indicated that R∆F NPs did not produce any behavioural changes and there was no clinical toxicity in mice treated with NPs compared to the non-treated group. These results were in line with the earlier studies (Yadav et al., 2022).

**TABLE 1:** Haematology and Coagulation Parameters in mice injected with R∆F NPs and PBS.

Name	RΔF	PBS	Statistics
Whole Blood Haemoglobin (gm/dL)	14.7 ± 1.12	14.67 ± 0.23	ns; *p* = 0.96
PCV/Haematocrit (%)	41.6 ± 3.53	40.33 ± 1.02	ns; *p* = 0.58
Total Leucocyte Count (10^∼9^/L)	3.46 ± 1.93	4.53 ± 2.06	ns; *p* = 0.62
Platelet Count (10^∼9^/L)	511 ± 62.22	582 ± 94.53	ns; *p* = 0.42
Neutrophils (%)	8 ± 3.60	9.6 ± 1.52	ns; *p* = 0.50
Lymphocytes (%)	91.33 ± 4.72	90 ± 2.30	ns; *p* = 0.54
Monocytes (%)	1 ± 0.57	1 ± 0	ns; *p* = 0.37
Eosinophils (%)	1 ± 0.57	0	

### 4.7 Antibody titre and *in vitro* parasite invasion inhibition

**FIGURE 5:**
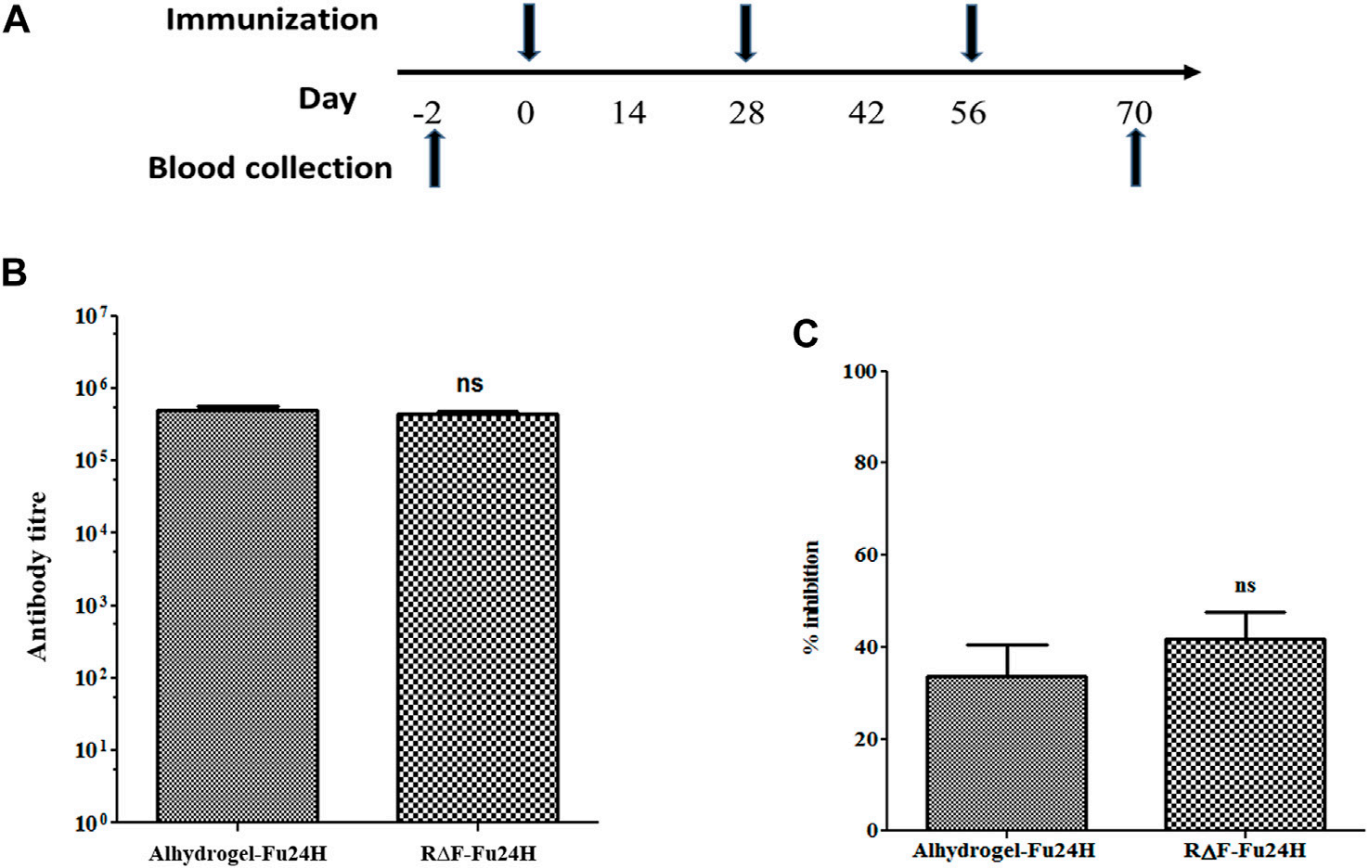
**(A)** A schematic drawing illustrating the immunization schedule for the R∆F-Fu24H complex and Alhydrogel^®^-Fu24H. **(B)** The end-point titers of antibodies against Fu24H were measured by ELISA. *p* > 0.05; not significant (Student’s t-test). **(C)** The inhibition of parasite invasion by anti-Fu24H antibodies was assessed against the *Plasmodium falciparum* laboratory line 3D7 parasite *in vitro*. P > 0.05; not significant (Student’s t-test).

Six BALB/c mice per group were given three intramuscular injections of 25 µg of Fu24H formulated with either R∆F NPs or Alhydrogel^®^ as an adjuvant as per the schedule shown in Figure 5A *.* Serum samples were collected prior to immunization and after the third immunization on day 70, and Fu24H-specific IgG titers were determined. Pre-immune sera from both groups showed no antibody titre to Fu24H. Fu24H-R∆F complex induced high Fu24H-specific IgG titers (4.3 × 10^5^), which is comparable to the antibody titre induced by FDA-approved adjuvant Alhydrogel®-Fu24H (4.9 × 10^5^) (*p* > 0.05 by Student’s t-test) (Figure 5B).

Next, we tested the invasion inhibitory activity of the antibodies present in the day 70 immune sera by performing a growth inhibition activity (GIA) assay on *P. falciparum* laboratory line 3D7. The GIA assay was used to compare the effectiveness of immune sera from R∆F-Fu24H complex and Alhydrogel®-Fu24H in blocking *P. falciparum* parasite invasion of erythrocytes. Figure 5C depicts the growth-inhibitory properties of the resulting antiserum. Immune sera from the R∆F-Fu24H complex and Alhydrogel®-Fu24H groups were able to inhibit erythrocyte invasion of *P. falciparum* by 40.1% and 37.8%, respectively, when tested at a 1:10 serum dilution. There was no statistically significant difference in the efficiency of inhibition by sera from R∆F-Fu24H complex and Alhydrogel®-Fu24H groups. These immunogenicity results suggest that R∆F NPs can effectively deliver antigens and elicit functional antibodies which partially inhibit parasite growth *in vitro*.

### 4.8 Cytokine response

**FIGURE 6:**
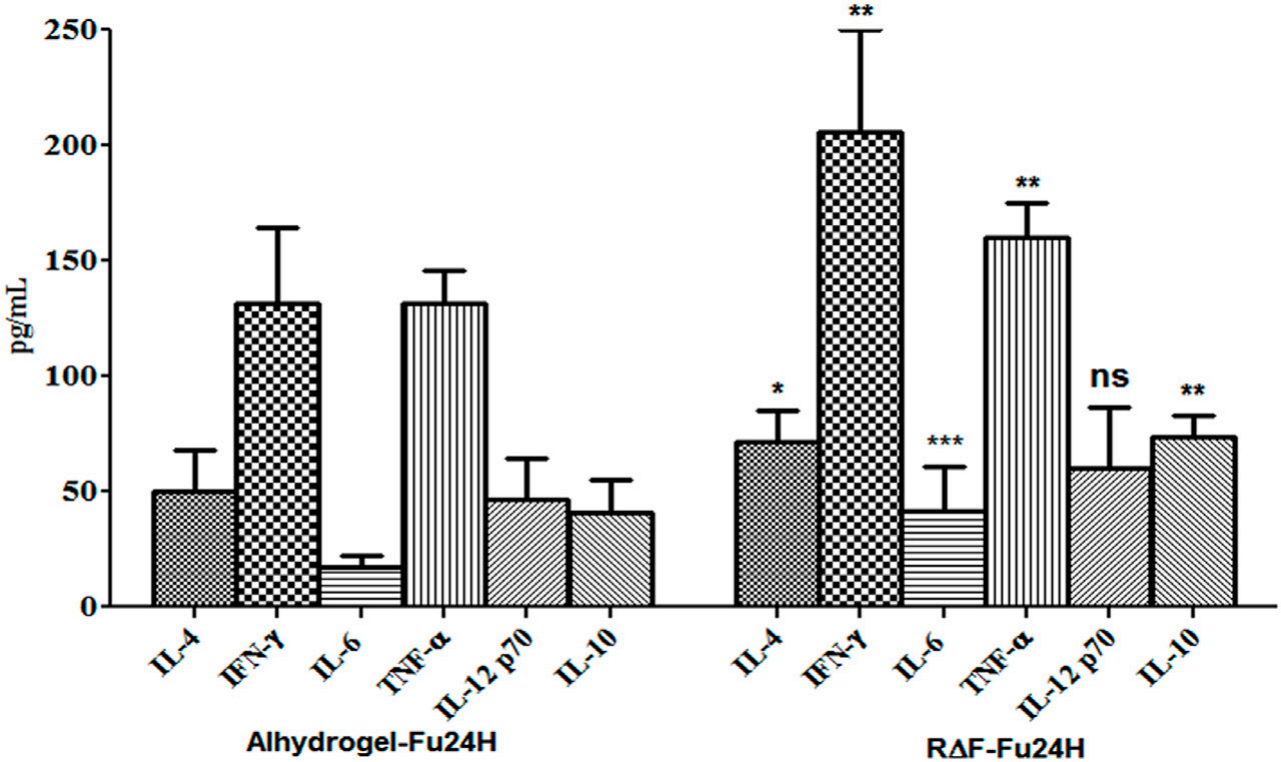
Cytokine response elicited by Fu24H delivered using R∆F NPs and Alhydrogel^®^. The amount of IL-4, IL-10, IFN-γ, TNF-ɑ, IL 6, and IL-12p70 elicited by Fu24H was measured using ELISA. Error bars represent mean ± standard deviation; *n* = 6 mice/group; Statistically significant (Student’s t-test; *p* < 0.01) data shown.

To determine the type of cytokine response induced by R∆F-Fu24H complex or Alhydrogel^®^-Fu24H, sera from day 70 post-immunization were tested for Th1 (IFN-γ, IL-12p70), Th2 (IL-4, IL-10), and inflammatory (IL-6, TNF-α) cytokines. Compared to Alhydrogel®-Fu24H, the R∆F-Fu24H complex induced significantly higher levels of IFN-γ (131.76 ± 32.75 pg/mL vs. 205.61 ± 44.26 pg/mL; *p* = 0.0082), TNF-α (131 ± 14.27 vs. 159.95 ± 15.18 pg/mL; *p* = 0.0079), IL-6 (17.01 ± 4.95 pg/mL vs. 41.39 ± 19.49 pg/mL; *p* = 0.004), IL-4 (49.98 ± 18.12 pg/mL vs. 71.84 ± 13.59 pg/mL; *p* = 0.039) and IL-10 (40.70 ± 14.08 pg/mL vs. 73.75 ± 9.28 pg/mL; *p* = 0.0038). There was no significant difference in the level of IL-12p70 (60.34 ± 26.31 pg/mL vs. 46.81 ± 17.27 pg/mL; *p* = 0.31) in mice immunized with Fu24H-R∆F complex or Alhydrogel®-Fu24H (Figure 6). These findings suggest that the cytokine response induced by the R∆F-Fu24H complex is comparable to that induced by Alhydrogel^®^.

## 5 Conclusions

Adjuvants are essential components of recombinant vaccines that stimulate desired immune responses. However, due to safety concerns and the materials’ origin, a limited number of licensed adjuvants are available for human use. Vaccine adjuvants should be non-toxic, capable of inducing sufficient immune responses, and biodegradable for possible clinical applications. Attention has been focused on nano-carriers, which offer a viable platform for presenting and activating a better immune response. We have demonstrated that R∆F NPs performed well as an antigen delivery system and immunostimulatory agent compared to the FDA-approved Alhydrogel^®^. Results showed that R∆F NPs are an appealing delivery platform because they are easy to produce and characterize, highly stable to proteolytic degradation, and can encapsulate antigen candidates efficiently and most importantly are highly biocompatible. Furthermore, recombinant Fu24H entrapped R∆F NPs induced high levels of anti-Fu24H specific antibody titres, showed *in vitro* invasion inhibitory activity and a mixed Th1/Th2 profile. These results clearly demonstrated that R∆F NPs might serve as a potential candidate for further development as a promising vaccine delivery vehicle system for clinical use. However, preclinical detailed toxicity, pharmacokinetic and pharmacodynamic studies for the nanoformulation have to be performed prior to regulatory approval and clinical use.

## Data Availability

The raw data supporting the conclusion of this article will be made available by the authors, without undue reservation.
