# Supplementary material for: Modified dipeptide based nanospheres as a potent adjuvating delivery system for recombinant vaccines
Source: Front Drug Deliv. 2023 Apr 26;3:1135209. doi: 10.3389/fddev.2023.1135209 (PMC12363250; doi:10.3389/fddev.2023.1135209)
Supplement: Supplementary file 1 [file DataSheet3.DOC]

**Modified dipeptide based nanospheres as a potent adjuvating delivery system of recombinant vaccines**

Saikat Biswas,^a^ Nitin Yadav,^a^ Anjali Somanathan,^b^ Paushali Mukherjee,^a,c^ Virander Singh Chauhan^a^*

* Corresponding Author

a. Malaria Research Group, International Centre for Genetic Engineering and Biotechnology, New Delhi, India.

b. School of Biotechnology, Jawaharlal Nehru University, New Delhi, India.

c. Multi Vaccines Development Program (MVDP), New Delhi, India.

**Address for Correspondence:**

Virander Singh Chauhan, Ph.D.

International Centre for Genetic Engineering and Biotechnology

Aruna Asaf Ali Marg

New Delhi-110067, India

Email: viranderschauhan@gmail.com

Telephone: +91-11-26741358

Fax: +91-11-26742316

**Keywords:** biomaterial, dipeptide, nano delivery, adjuvant, malaria, recombinant vaccine, humoral and cellular response

**Supplementary text**

**Text S1:** Briefly, Boc-Arg(pbf)-OH (5 mM) was dissolved in dry THF, chilled to -20^o^ C, and stirred in an ice-salt bath. Next, equivalent moles of NMM and IBCF was added and left for 10 minutes. Pre-cooled aqueous solutions of DL-3- Phenylalanine hydrate (5.5 mM) and sodium hydroxide (5.5 mM) were then added and stirred overnight. The obtained residue was acidified with citric acid and extracted with ethyl acetate. The ethyl acetate layer was washed thrice with water and saturated sodium chloride. It was then dried over anhydrous sodium sulfate and evaporated to get Boc-Arg(pbf)-DL-3-Phenylalanine, which was next mixed with anhydrous sodium acetate (5.5 mM) in freshly distilled acetic anhydride and 1.1 molar excess of anhydrous sodium acetate (0.9 gm). The slurry was poured over crushed ice and stirred continuously till a yellow-colored precipitate was formed. The residue was filtered through a grade 4 sintered funnel and washed with 5% sodium bicarbonate solution and cold water. The azlactone (Azl), Boc-Arg(pbf)-∆Phe-Azl was dried completely using a desiccator. The resulting compound was dissolved in methanol and treated with 1.5 equivalent of 1 N NaOH solution for 3 - 4 hours. The obtained residue was acidified with citric acid and extracted with ethyl acetate. The ethyl acetate layer was washed thrice with water and saturated sodium chloride. The Boc group was deprotected using TFA.

**Supplementary Figures:**


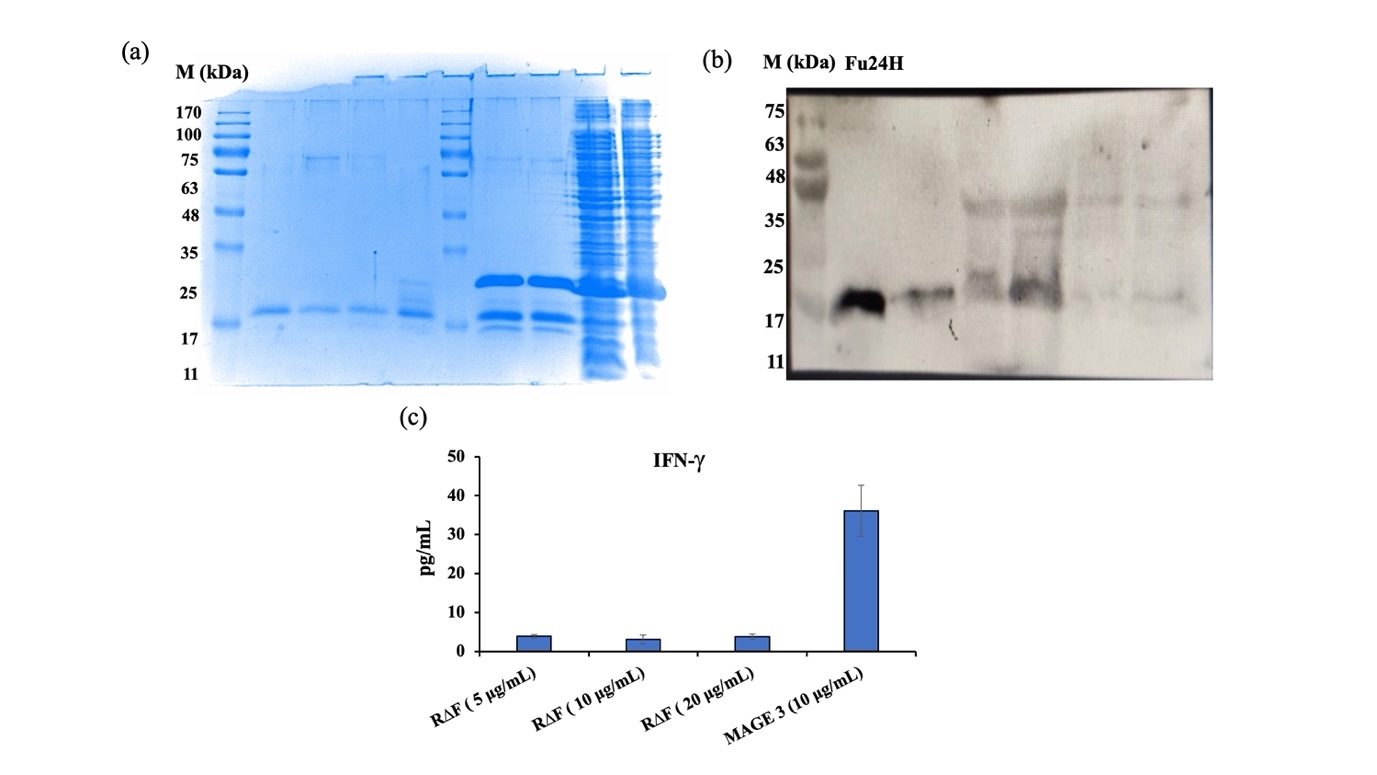


**Figure S1.** (a) Uncropped 15% SDS-PAGE of Fu24H. (b) Uncropped western blot of Fu24H. (c) IFN-γ response in *in vitro* co-culture together with DCs plus T cells in the presence of RΔF NPs and positive control MAGE 3.


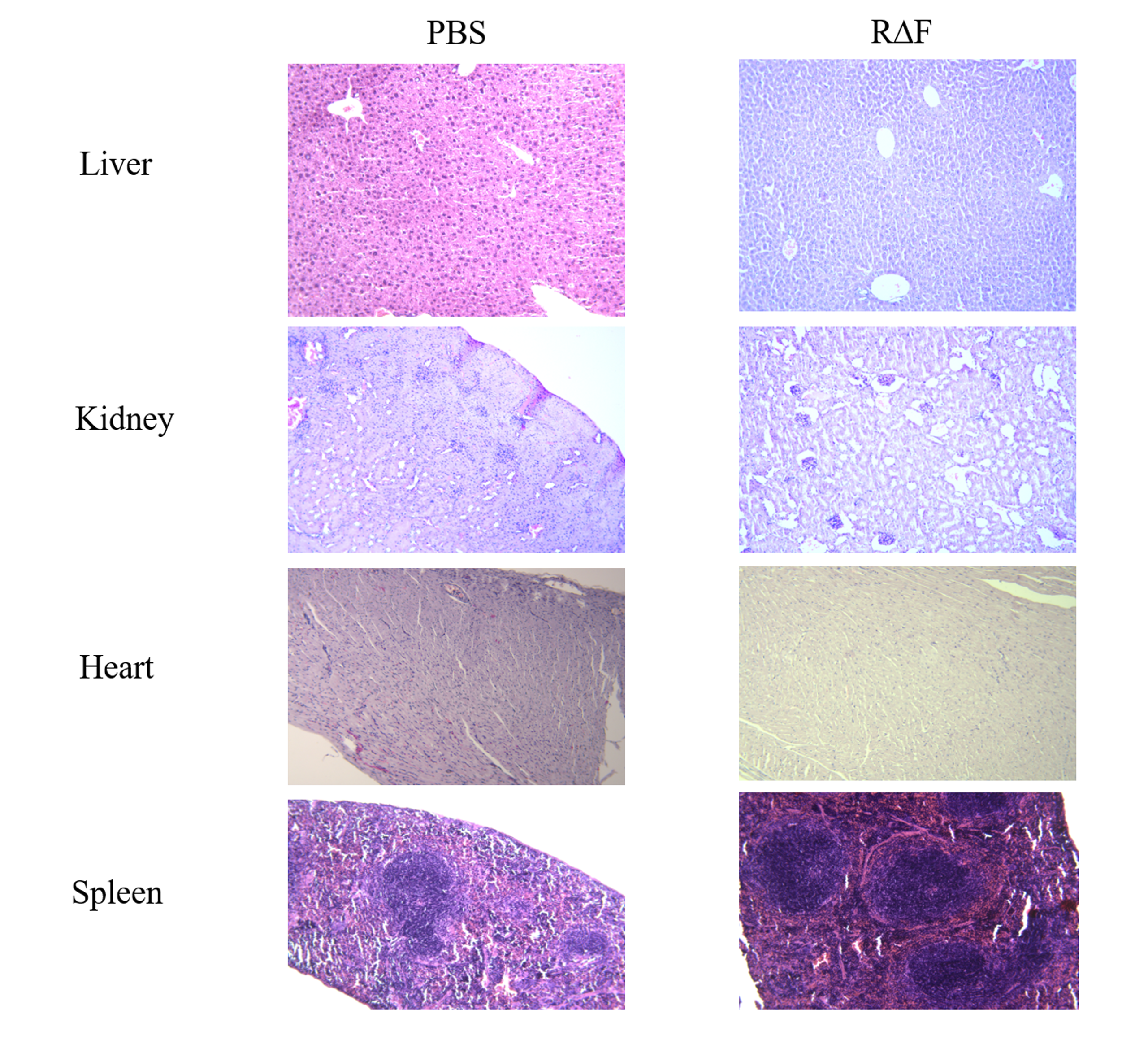


**Figure S2:** Histopathological examination using hematoxylin and eosin (H&E) staining for major organs, liver, kidney, spleen, and heart. Images were captured at 10X magnification.
